# (COVID-19) SARS-CoV-2-catatonia syndrome: does it exist? Longitudinal evidence

**DOI:** 10.3389/fpsyt.2026.1755846

**Published:** 2026-03-13

**Authors:** Diğdem Göverti, Elif Poyraz, Bestenur Güvendi Melenkiş, Duygu Nur Tutam, Serdar M. Dursun

**Affiliations:** 1 Department of Psychiatry, Kocaeli University Faculty of Medicine, Kocaeli, Türkiye; 2 Department of Psychiatry, Iğdır Dr. Nevruz Erez State Hospital, Iğdır, Türkiye; 3 Department of Psychiatry, Erenköy Training and Research Hospital for Mental Health and Neurological Disorders, Istanbul, Türkiye; 4 Department of Psychiatry, University of Health Sciences, Van Training and Research Hospital, Van, Türkiye; 5 Department of Psychiatry, University of Alberta, Edmonton, AB, Canada

**Keywords:** catalepsy, catatonia, COVID-19, mutism, negativism, SARS-CoV-2

## Abstract

**Introduction:**

Catatonia is a complex neurobehavioral syndrome related with several psychiatric and medical conditions. The underlying pathophysiological mechanism is the dysfunction of cortical-subcortical motor regulation systems, including GABA, dopamine, and glutamate, or increased and sympathetic freezing response may be some of the mechanisms. The pathogenesis of neuropsychiatric complications related to COVID-19 is associated with nervous system damage due to systemic inflammation and cytokine storm. In catatonia, evidence of acute phase activation has been shown rarely. In this study, we aimed to investigate the relationship between COVID-19 and catatonia, which has been reported as case reports in the literature.

**Materials and methods:**

This study has been designed as a retrospective chart review. The data was collected by three psychiatrists for the period of four years, separated into before and after the released first case (11/03/2020) from emergency psychiatry department, outpatient and inpatient clinics of Erenköy Training and Research Hospital for Mental Health and Neurological Disorders. The keywords when searching in the hospital database were “catatonia”, “catatonia-syndrome”, and all catatonia symptoms in DSM-5. After the pandemia started, we searched for COVID-19 infection additionally.

**Results:**

Forty patients were included in the study, consisting of 20 females (50.00%) and 20 males (50.00%). There was no significant difference in pre-(n:18) and post-COVID 19 (n:22) cases according to age, gender, underlying cause, treatment applied in two groups (p<0.05). In addition, symptom diversity of catatonia was not statistically significant in both groups (p<0.05). In the post-pandemic period, 2 cases were diagnosed with COVID-19 in the last month.

**Discussion:**

Postinfectious COVID-19 catatonia is a rare neuropsychiatric complication of COVID-19. The result of our study supports the unclear role of acute phase reactants in catatonia. Neuropsychiatric symptomatology is broad in postinfectious COVID-19. As understanding of the pathogenesis remains fairly limited, symptomatic management is an appropriate strategy.

## 1 Introduction

Catatonia is a psychomotor syndrome characterized by motor abnormalities that include excessive movements, reduced mobility, abnormal movements, mutism or abnormalities of speech, negativism and reduced functioning (1). It is a complex syndrome that can arise from serious psychiatric disorders and medical causes, and its pathophysiology remains unclear (2). Within the catatonic presentation, nearly 40 symptoms and signs can be observed in various combinations (3). In recent years, data suggesting a decrease in the frequency of catatonia with the development of new treatment methods and indicating its absence in developed countries have emerged; however, these findings have not been confirmed (3, 4). Studies related to catatonia have determined its frequency in psychiatric illnesses to be around 10–13% (1).

The term *catatonia* derives from the Greek words *kata* and *tonos*, meaning “down” and “tension” (3). It was first systematically described by Kahlbaum in 1874, based on 26 cases with stuporous, excited, and malignant psychomotor features, and conceptualized as a distinct brain disorder with a cyclic and progressive course (5). In 1913, Kraepelin classified catatonia under *dementia praecox*, a view that shaped psychiatric nosology for decades. Until DSM-5 (2013), catatonia was largely considered a subtype of schizophrenia. DSM-IV (1994) introduced “catatonia due to a general medical condition,” and DSM-5 redefined it as a specifier applicable to psychotic, mood, and medical or neurological disorders (6, 7). In ICD-10, catatonia was categorized under schizophrenia; however, ICD-11 now defines it as a separate diagnostic entity. This change acknowledges its transdiagnostic significance, as it may present in numerous psychiatric and medical disorders beyond schizophrenia (5).

Catatonia can present in various clinical manifestations. Findings and symptoms associated with catatonia can be grouped into four clusters: pure motor signs (posturing, rigidity, akinesia), disorders of will (ambivalence, negativism, command automatism), inability to suppress complex motor movements (stereotypies, echolalia, echopraxia), and autonomic instability (tachycardia, high fever). The prognosis worsens when autonomic symptoms are added to catatonic symptoms, and it is referred to as “malignant catatonia’’ (6, 7). According to DSM-5 (2013), diagnosis can be made without a duration criterion, with the presence of 3 or more catatonic symptoms. In studies where catatonia assessment scales were used, the prevalence rates of catatonia were found to be higher than those reported in research conducted with DSM criteria (8). This allows us to assert that the DSM criteria may be restrictive.

Catatonia has been described as a feature of psychiatric diagnoses such as psychotic disorders, mood disorders and autism (7, 9, 10), as well as in various conditions such as viral infections, autoimmune disorders or metabolic disturbances (11). In studies, it has been demonstrated that 10–15% of catatonic patients meet the criteria for schizophrenia. Additionally, in manic patients, 25% or more exhibit catatonic symptoms at a level that would warrant a diagnosis of catatonia according to DSM criteria (7).

The neurobiological mechanism of catatonia is not fully understood; however, the positive response to benzodiazepines and worsening with dopamine antagonists suggest the involvement of GABAergic and dopaminergic alterations (12). Supporting this, brain imaging has shown decreased GABA-A density in the left sensorimotor cortex (13). Although some studies indicate lorazepam is not superior to placebo, it remains a diagnostic and therapeutic tool via the lorazepam challenge test (LCT) (14). Furthermore, the effectiveness of ECT suggests it may restore impaired intracortical inhibition by modulating GABAergic neurotransmission (15). While antidopaminergic drugs exacerbate symptoms, the lack of efficacy of dopaminergic agents indicates that dopamine’s role remains partially explained (6). Additionally, the description of anti-NMDA receptor encephalitis (16) and the positive outcomes observed with amantadine (17) highlight the involvement of the glutamate system. Collectively, dysfunction in cortical-subcortical motor regulation—encompassing GABA, dopamine, and glutamate—may represent a biological vulnerability (12).

Infectious and autoimmune diseases, including CNS infections and autoimmune encephalitis, are also known to trigger catatonia through direct brain impact or neuroinflammation mediated by proinflammatory cytokines (2). Recently, various COVID-19-associated catatonia cases have been reported (2, 18, 19), including “delayed” or “excited” presentations occurring weeks after infection (20, 21). Beyond direct viral effects, pandemic-related restrictions may have influenced catatonia frequency through treatment interruptions or the psychological impact of quarantine (22, 23). As the relationship between COVID-19 and catatonia remains unclear, this study aims to compare the frequency and clinical distribution of catatonic symptoms in a psychiatric center before and after the pandemic period.

## 2 Materials and methods

### 2.1 Sample and data extraction

The data was collected by three psychiatrists for the period of four years (2018–2022), separated into before and after the released first case (11/03/2020) in Türkiye. The patients were selected from the emergency department, outpatient clinic, and inpatient services of the University of Health Sciences, Erenkoy Mental Health and Neurological Disorders Training and Research Hospital. COVID-19 infections occurring within two weeks prior to the onset of catatonic symptoms were considered positive, in line with the period defined by health authorities for COVID-19 recovery. The post-pandemic group consisted of consecutive catatonic patients regardless of recent COVID-19 PCR results, focusing on the overall pandemic-era incidence rather than acute viral manifestations The inclusion criteria for the study were being 18 years of age or older and having accessible clinical data. The exclusion criteria included the inability to access medical data for any reason and having received a diagnosis of catatonia due to the general medical condition. To exclude underlying medical conditions, general physical examination, cranial CT, inflammatory markers, routine blood tests, liver and renal function tests, and electrolyte levels were evaluated. A retrospective data analysis was conducted using the hospital computer system, and 40 patients meeting the inclusion criteria were included in the study. This retrospective study was approved by the local ethics committee of Erenkoy Mental Health and Neurologic Disorders Training and Research Hospital.

### 2.2 Assessment tools

#### 2.2.1 Sociodemographic and clinical information form

With the form prepared by the researcher, the sociodemographic information of the patient, clinical details regarding the psychiatric disease process, and data pertaining to the history of COVID-19 infection have been recorded.

#### 2.2.2 Bush-Francis Catatonia Rating Scale

The Bush-Francis Catatonia Rating Scale (BFCRS), one of the most widely used scales in the assessment of catatonic symptoms, was developed in 1996 and consists of 23 items. The initial 14 items in this scale are also used for screening purposes. Some of the symptoms and signs are rated on a scale of 0-3, while others are classified as present/absent (24).

#### 2.2.3 DSM-5 catatonia diagnostic criteria

In DSM-5, the diagnostic criteria for catatonia include 12 symptoms: catalepsy, waxy flexibility, stupor, agitation, mutism, negativism, posturing, mannerisms, stereotypies, grimacing, echolalia, and echopraxia. It is indicated that for a diagnosis of catatonia, at least three of these 12 symptoms should be present.

### 2.3 Statistical analyses

The statistical analysis of the study was performed using Statistical Package for the Social Sciences (SPSS) version 25. The adequacy of the normal distribution for the variables was examined through histogram graphs and the Kolmogorov-Smirnov test. Descriptive analyses were presented using mean, standard deviation, median, and minimum-maximum values. In 2x2 tables, comparisons were made using Pearson’s Chi-Square and Fisher’s Exact Tests. For variables that did not exhibit a normal distribution (nonparametric), the Mann-Whitney U Test was employed to evaluate differences between groups. A significance level of p < 0.05 was considered statistically significant in all comparisons.

## 3 Results

Forty patients were included in the study, consisting of 20 females (50.00%) and 20 males (50.00%). The mean age of the participants was 40.18 ± 13.37 years. It was observed that 17 individuals had completed primary school (42.00%), 10 individuals (25.00%) were high school graduates, and the majority were single (n=26, 65.00%). 28 patients (70%) were smokers, 12 patients (30.77%) were alcohol consumers, while one person (2.50%) reported substance use. When psychiatric diagnoses were examined, 40% of the participants (n=16) had schizophrenia, 25% (n=10) had depressive disorder, and 22.50% (n=9) were diagnosed with bipolar disorder. 12 individuals (30.00%) had additional medical conditions. Fourteen individuals (35.00%) received psychiatric treatment before admission, and electroconvulsive therapy (ECT) was administered to 13 individuals (32.50%) during hospitalization. When examining the dates of hospital admission, 18 patients (45.00%) applied before the pandemic, while 22 patients (55.00%) applied after the pandemic.

Among the post-pandemic cases, two patients were diagnosed with COVID-19 within one month. However, these cases fell outside the predefined two-week time frame and were therefore excluded from the analysis. The limited number of such cases precluded statistical evaluation (**Table 1**).

**Table 1:** Comparison of pre and post-pandemic sociodemographics.

Variables	Subgroups	Pre-pandemic		Post-pandemic		P
		n/Mean±SD	%/Median (Min-Max)	n/Mean±SD	%/Median (Min-Max)	
Sex	Female	9	(50,00)	11	(50,00)	1,000
	Male	9	(50,00)	11	(50,00)	
Age		40,06±11,9	38,5 (24-69)	40,27±14,75	34,5 (23-68)	0,840¹
Marital status	Married	7	(38,89)	7	(31,82)	0,641
	Single	11	(61,11)	15	(68,18)	
Educational level	Elementary school	10	(55,56)	7	(31,82)	0,574
	High school	3	(16,67)	7	(31,82)	
	University	3	(16,67)	4	(18,18)	
	Master’s degree	1	(5,56)	1	(4,55)	
	None	1	(5,56)	3	(13,64)	
Employment status	Employed	2	(11,11)	4	(18,18)	0,533
	Unemployed	16	(88,89)	18	(81,82)	
Cigarette smoking	Smoker	13	(72,22)	15	(68,18)	0,781
	Non-smoker	5	(27,78)	7	(31,82)	
Pack/years of smoking		21,08±11,55	20 (7-40)	18,8±12,23	15 (5-42)	0,586¹
Alcohol use	Absent	10	(58,82)	17	(77,27)	0,216
	Social drinking	7	(41,18)	5	(22,73)	
Substance use	Present	1	(5,56)	0	(,00)	0,263
	Absent	17	(94,44)	22	(100,00)	
Psychiatric diagnosis	Schizophrenia	8	(44,44)	8	(36,36)	0,891
	Bipolar disorder	4	(22,22)	5	(22,73)	
	Depressive disorder	4	(22,22)	6	(27,27)	
	Schizoaffective dis	2	(11,11)	2	(9,09)	
	Other	0	(,00)	1	(4,55)	
Additional medical condition	Present	5	(27,78)	7	(31,82)	0,781
	Absent	13	(72,22)	15	(68,18)	
Pre-admission psychiatric treatment	Present	7	(38,89)	7	(31,82)	0,641
	Absent	11	(61,11)	15	(68,18)	
ECT during hospitalization	Yes	7	(38,89)	6	(27,27)	0,435
	No	11	(61,11)	16	(72,73)	
Number of ECT sessions		7,71±2,93	7 (5-14)	7,67±1,63	7,5 (6-10)	0,731¹
History of having had a COVID-19 infection	Yes	0	(,00)	2	(9,52)	***
No	0	(,00)	19	(90,48)		

Chi-square Test ¹and Mann-Whitney U Test.

*** P-value could not be calculated because the variable was constant (zero) in the pre-pandemic group.

**Figure 1:**
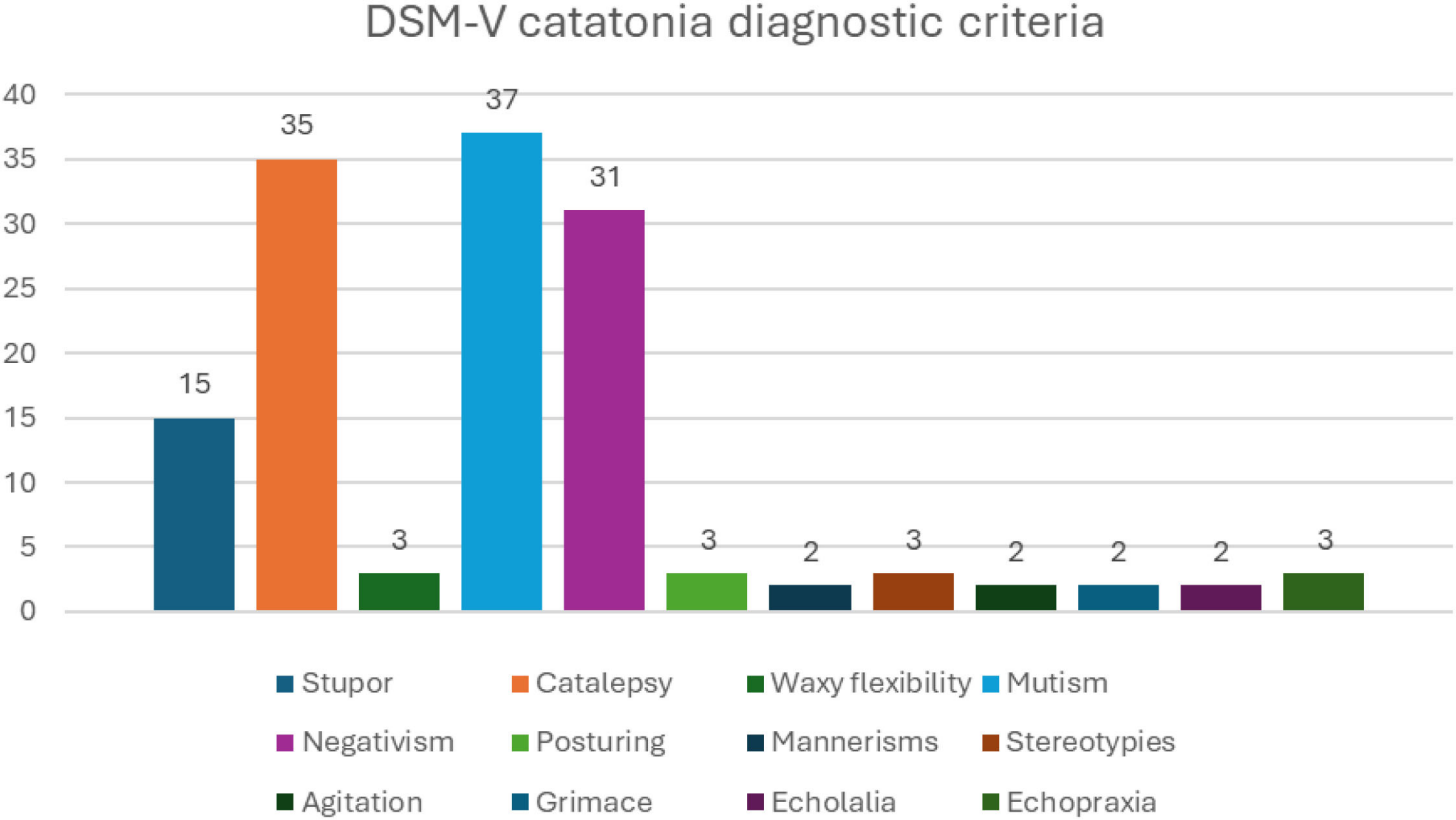
Catatonia symptom distribution.

When examining catatonic symptoms in participants (n=40) according to the DSM-5 catatonia diagnostic criteria, mutism was observed in 37 patients (92.50%), catalepsy in 35 (87.50%), and negativism in 31 (77.50%). The least observed symptoms were mannerisms, agitation, grimacing, and echolalia (**Figure 1**).

The distribution and scores of the Bush-Francis Catatonia Scale were calculated. When examining the Bush-Francis Catatonia Scale scores of participants (n=40), the mean total score was found to be 10.3 ± 2.53. Echopraxia/echolalia, verbigeration, automatic obedience, mitgehen, gegenhalten, ambitendency, perseveration, combativeness, and autonomic abnormality symptoms and signs were not observed (**Table 2**).

**Table 2:** Comparison of Bush-Francis Catatonia Scale score in pre and post-pandemic terms.

Variables	Clinical Presentation	Pre-pandemic		Post-pandemic		P
		n/Mean±SD	%/Median (Min-Max)	n/Mean±SD	%/Median (Min-Max)	
Excitement	Absent	15	(83,33)	16	(72,73)	0,564
	Intermittent	3	(16,67)	5	(22,73)	
	Constant	0	(,00)	1	(4,55)	
Immobility/stupor	Absent	4	(22,22)	2	(9,09)	0,163
	Brief interact	2	(11,11)	8	(36,36)	
	No interaction	9	(50,00)	11	(50,00)	
	Stuporous	3	(16,67)	1	(4,55)	
Mutism	Absent	1	(5,56)	0	(,00)	0,183
	Whisper	0	(,00)	3	(13,64)	
	Less than 20 words/5 min	6	(33,33)	10	(45,45)	
	No speech	11	(61,11)	9	(40,91)	
Staring	Absent	15	(83,33)	18	(81,82)	0,670
	Poor eye contact	1	(5,56)	2	(9,09)	
	Gaze held longer than 20 s	2	(11,11)	1	(4,55)	
	Fixed gaze, non-reactive	0	(,00)	1	(4,55)	
Posturing/catalepsy	Absent	6	(33,33)	4	(18,18)	0,400
	Less than 1 min	1	(5,56)	5	(22,73)	
	Between 1 and 15 min	7	(38,89)	9	(40,91)	
	Bizarre posture or more than 15 min	4	(22,22)	4	(18,18)	
Grimacing	Absent	17	(94,44)	21	(95,45)	0,360
	Less than 1 min	0	(,00)	1	(4,55)	
	Bizarre expression or more than 1 min	1	(5,56)	0	(,00)	
Stereotypy	Absent	17	(94,44)	20	(90,91)	0,653
	Occasional	0	(,00)	1	(4,55)	
	Frequent	1	(5,56)	1	(4,55)	
Mannerisms	Absent	18	(100,00)	21	(95,45)	0,360
	Occasional	0	(,00)	1	(4,55)	
Rigidity	Absent	16	(88,89)	20	(90,91)	0,499
	Mild resistance	0	(,00)	1	(4,55)	
	Moderate	2	(11,11)	1	(4,55)	
Negativism	Absent	2	(11,11)	2	(9,09)	0,579
	Mild resistance	4	(22,22)	9	(40,91)	
	Moderate resistance	5	(27,78)	6	(27,27)	
	Severe resistance	7	(38,89)	5	(22,73)	
Waxy flexibility	Absent	15	(83,33)	22	(100,00)	0,083*
	Present	3	(16,67)	0	(,00)	
Withdrawal	Absent	4	(22,22)	1	(4,55)	0,397
	Minimal PO less than 1 day	2	(11,11)	4	(18,18)	
	Minimal PO more than 1 day	9	(50,00)	13	(59,09)	
	No PO	3	(16,67)	4	(18,18)	
Impulsivity	Absent	17	(94,44)	20	(90,91)	0,653
	Occasional	1	(5,56)	1	(4,55)	
	Frequent	0	(,00)	1	(4,55)	
Grasp reflex	Absent	17	(94,44)	22	(100,00)	0,450*
	Present	1	(5,56)	0	(,00)	
BFCS total score		10,44±2,83	10 (6-18)	10,18±2,32	10 (7-15)	0,925¹

Chi-square Test *Fisher’s Exact Test ¹Mann Whitney-U Test.

The patients admitted to the hospital before and after the pandemic were compared in terms of DSM-5 catatonia diagnostic criteria, and no statistically significant difference was found between the two groups.

Symptom presentations according to DSM-5 were compared between the pre-pandemic and post-pandemic periods. No statistically significant difference was observed between the two groups.

Mutism emerged as the most prevalent symptom across both study periods. While a numerical decrease in the severity of the “no speech” category was observed in the post-pandemic group (P = 0.183), its overall clinical prevalence remained stable. Similarly, the frequency of negativism according to DSM-5 criteria remained nearly constant (P = 0.636), and although the proportion of patients exhibiting “severe resistance” on the BFCRS decreased numerically, this change did not reach statistical significance (P = 0.579). Regarding stupor, while DSM-5 frequencies showed a downward trend post-pandemic, the results were not statistically significant (P = 0.517). However, a notable decrease in “stuporous” cases was recorded in the BFCRS table, suggesting that stupor may have presented in milder forms in the post-pandemic cohort. Finally, waxy flexibility was identified as one of the rarest symptoms; according to both DSM-5 and BFCRS data, this symptom completely disappeared in the post-pandemic group (P = 0.083).

## 4 Discussion

This study aimed to evaluate the impact of the COVID-19 pandemic on the clinical presentation and incidence of catatonia. In our sample, 18 cases of catatonia were identified during the two years preceding March 11, 2020—the date of the first confirmed COVID-19 case in Turkey—and 22 cases were identified during the subsequent two years. Based on these data, we cannot conclude that COVID-19 infection led to an increase in catatonia cases. We compared catatonic patients before and after the pandemic in terms of sociodemographic and clinical characteristics, but no significant differences were observed. The results obtained in this section were interpreted in light of the existing literature.

The mechanisms underlying COVID-19 remain unclear in the cases reported to date (23). The neuropsychiatric manifestations associated with the infection constitute a significant area of investigation. Previous pandemics have been accompanied by a range of neuropsychiatric symptoms—including acute and late-stage encephalopathy, demyelinating diseases, psychosis, and mood disorders—although the mechanisms continue to be debated. Both the severe psychosocial stress induced by the pandemic and the direct or indirect effects of the infection on the central nervous system are thought to contribute to various neuropsychiatric symptoms. These symptoms span a broad spectrum, from headaches, anxiety, and sleep disturbances to potentially life-threatening conditions such as catatonia (2, 23).

Most studies examining the association between COVID-19 and catatonia consist of case series and review articles (18–23). The effect of COVID-19 on the incidence of catatonia therefore remains unclear, underscoring the need for further research. In this regard, our study contributes important preliminary data. However, we found no significant difference between the number of catatonia cases identified before and after the pandemic.

Several factors may account for these findings. One crucial point regarding catatonia is that it is a diagnosis frequently overlooked in clinical practice. Similarly, Llesuy et al. reviewed inpatient medical records and found that 79 of 133 patients who met DSM-5 criteria for catatonia were not diagnosed at the time of admission (25). Their study emphasized that psychiatric consultation played a critical role in reducing the likelihood of missed diagnoses.

In our study, catatonic patients before and after the pandemic displayed similar sociodemographic characteristics, including age, gender, marital status, education level, and employment status. Half of the patients were women, and the mean age was 40.18 years. In a review by Dawood et al. (26) of 42 catatonic COVID-19 cases across 27 studies, no gender differences were observed, and the age distribution tended to cluster above 50 years.

Previous studies have suggested that older age, comorbid medical conditions, and a history of psychiatric illness may predispose individuals to developing catatonia during COVID-19 infection (23). Our study did not include patients with catatonia secondary to a general medical condition; only psychiatric patients were assessed. Catatonia is known to be most frequently associated with mood disorders, particularly mania (12). Although 30% of our sample had comorbid medical illnesses, half were diagnosed with psychotic disorders and 47.5% with mood disorders. Dawood et al. (26) similarly reported that 24% of patients had no prior psychiatric or medical illness.

Furthermore, catatonia is established as a discrete clinical entity occurring across diverse psychiatric and medical contexts; thus, its diagnostic criteria and therapeutic interventions remain uniform irrespective of the primary cause. This independent nature of the syndrome likely accounts for the lack of statistical significance between the pre- and post-pandemic eras in our study, notwithstanding the presence of COVID-19 cases (27). As illustrated in **Table 3**, a downward trend was observed in the frequency of the most common symptoms, such as mutism and negativism, in the post-pandemic period, which was more pronounced in the BFCRS data compared to DSM-5 (**Table 4**). This discrepancy can be attributed to the methodological nature of the BFCRS; while DSM-5 utilizes a categorical ‘present/absent’ approach, the BFCRS employs a four-point rating scale for each symptom. This grading system allows for a more sensitive detection of nuances in symptomatic severity and subtle clinical improvements that might be overlooked by the binary classification of DSM-5 (28).

**Table 3:** Comparative summary table (pre-pandemic *vs*. post-pandemic).

Symptom	DSM-5 frequency (Pre vs Post)	BFCRS severity (Pre vs Post)
Mutism	Pre: 94.44% Post: 90.91%	Pre: 61.11% (No speech) Post: 40.91% (No speech)
Negativism	Pre: 77.78% Post: 77.27%	Pre: 38.89% (Severe resistance) Post: 22.73% (Severe resistance)
Stupor	Pre: 44.44% Post: 31.82%	Pre: 16.67% (Stuporous) Post: 4.55% (Stuporous)
Waxy Flexibility	Pre: 11.11% Post: 0.00%	Pre: 16.67% (Present) Post: 0.00% (Absent)

**Table 4:** Comparison of catatonia symptoms in pre and post-pandemic terms.

Variables	Status	Pre-pandemic		Post-pandemic		P
		n	%	n	%	
Stupor	Present	8	(44,44)	7	(31,82)	0,517
	Absent	10	(55,56)	15	(68,18)	
Catalepsy	Present	15	(83,33)	20	(90,91)	0,642
	Absent	3	(16,67)	2	(9,09)	
Waxy flexibility	Present	3	(11,11)	0	(4,55)	0,579
	Absent	15	(88,89)	22	(95,45)	
Mutism	Present	17	(94,44)	20	(90,91)	0,577
	Absent	1	(5,56)	2	(9,09)	
Negativism	Present	14	(77,78)	17	(77,27)	0,636
	Absent	4	(22,22)	5	(22,73)	
Posturing	Present	3	(16,67)	0	(,00)	0,083
	Absent	15	(83,33)	22	(100,00)	
Mannerisms	Present	0	(,00)	2	(9,09)	0,492
	Absent	18	(100,00)	20	(90,91)	
Stereotypies	Present	0	(,00)	3	(13,64)	0,238
	Absent	18	(100,00)	19	(86,36)	
Agitation	Present	1	(5,56)	1	(4,55)	0,704
	Absent	17	(94,44)	21	(95,45)	
Grimace	Present	1	(5,56)	1	(4,55)	0,704
	Absent	17	(94,44)	21	(95,45)	
Echolalia	Present	1	(5,56)	1	(4,55)	0,704
	Absent	17	(94,44)	21	(95,45)	
Echopraxia	Present	1	(5,56)	2	(9,09)	0,577
	Absent	17	(94,44)	20	(90,91)	

Fisher’s Exact Test.

In situations where access to healthcare is disrupted, such as during a pandemic, chronic psychiatric patients may experience treatment interruptions and symptom exacerbations (11). Of the 40 catatonic patients in our study, 26 were not taking psychiatric medication before admission. Nonetheless, there was no significant difference between pre- and post-pandemic groups in terms of psychiatric treatment status prior to hospitalization.

In our sample, the mean interval between COVID-19 infection and hospitalization among post-pandemic catatonic patients was 15 days. Although most reported cases in the literature describe catatonia emerging during active infection, there are also accounts of symptoms developing weeks after recovery. We identified two patients who had been diagnosed with COVID-19 one month prior to the onset of catatonic symptoms, rather than within 15 days. These cases, identified descriptively, may represent examples of delayed or excited catatonia as reported in the literature. However, unlike previously published cases, these patients no longer exhibited active infection symptoms and were no longer COVID-19 positive at the time of catatonia onset, which distinguishes them from earlier reports (20, 21).

According to DSM-5 criteria, the most common symptoms observed in our sample were mutism, catalepsy, and negativism—findings consistent with the literature (11). Llesuy et al. (25) identified agitation, grimacing, and echolalia as risk factors for missed catatonia diagnoses, as these symptoms may be less readily associated with the condition. In our study, these symptoms were also among the least frequently observed.

Although the body of knowledge on COVID-19 continues to expand, many aspects of the infection remain incompletely understood. COVID-19 has been associated with a range of neuropsychiatric manifestations in both the acute and post-acute phases, and catatonic symptoms may be considered among the possible clinical presentations reported in this context. While our study provides preliminary observations, further research is needed to clarify any potential relationship between COVID-19 and the incidence or clinical features of catatonia.

The cohort design and the use of two different rating scales to assess catatonic symptoms constitute strengths of our study. However, several limitations should be acknowledged. First, the retrospective design and incomplete records may have affected data quality. Second, the study was conducted in a single mental health center with a 200-bed capacity, which may limit the generalizability of the findings. Although the data were obtained from the second largest mental health hospital in Istanbul, the sample size remained relatively small. In addition, the study was conducted in a mental health hospital that admits only psychiatric inpatients, which may have contributed to the underdiagnosis or oversight of COVID-19 infections. Finally, patient assessments and the application of rating scales were performed by different clinicians, which may have introduced inter-rater variability.

## 5 Conclusion

In conclusion, although the association between COVID-19 and catatonia remains unclear, our retrospective study did not identify a statistically significant difference in case numbers before and after the COVID-19 pandemic. By systematically examining this relationship beyond isolated case reports, our findings contribute to the existing literature. Additionally, no differences in clinical presentation were observed, irrespective of etiopathogenetic considerations.

## Data Availability

The original contributions presented in the study are included in the article/supplementary material. Further inquiries can be directed to the corresponding author.
